# Assessing Plant-Based Diets in Taiwan Using a Harmonized Food Description-Incorporated Framework

**DOI:** 10.3390/nu17142268

**Published:** 2025-07-09

**Authors:** Yu-Syuan Wei, Ming-Hua Lin, Fu-Jun Chen, She-Yu Chiu

**Affiliations:** 1 Center for Biomedical Resources, National Health Research Institutes, Miaoli 35053, Taiwan; msyswei@gmail.com; 2 Center for General Education, National Formosa University, Yunlin 63201, Taiwan; minhua5356@gmail.com; 3 Centers for Disease Control, Ministry of Health and Welfare, Taipei 10050, Taiwan; jfchen@cdc.gov.tw; 4 Institute of Population Health Sciences, National Health Research Institutes, Miaoli 35053, Taiwan

**Keywords:** food classification and description system, plant-based diet, nutrient-rich food index, dietary quality, HFDFC system

## Abstract

**Background**: Exploring emerging dietary patterns, such as plant-based diets (PBD), often requires considerable effort to rebuild new systems or adapt existing food classification frameworks, presenting a substantial challenge for dietary research. Current systems were not originally designed for this purpose and vary in standardization and interoperability, complicating cross-study comparisons. This study aimed to adopt the harmonized, food description-incorporated, food classification system (HFDFC system) to develop a plant-based diet food classification system (PBDFC system), and to evaluate dietary intake and nutritional status among adults in Taiwan. **Methods**: A repeated cross-sectional design was applied using 24 h dietary recall data from the Nutrition and Health Survey in Taiwan (2013–2016 and 2017–2020), accessed via the national food consumption database. Adults aged 20–70 years were included. Data were processed through the HFDFC system to generate the PBDFC system. For each participant, the Plant-Based Diet Index (PDI), Body Mass Index (BMI), and Nutrient-Rich Food Index (NRF) were calculated and analyzed by age group. **Results**: Adults aged 46–70 had significantly higher O-PDI and H-PDI scores, lower Lh-PDI scores (all *p* < 0.0001), and higher NRF values. Despite higher average BMI, those in the highest H-PDI tertile had significantly lower BMI (*p* < 0.02). **Conclusions**: The HFDFC-based PBDFC system offers a flexible, scalable framework for plant-based diet classification and supports future cross-national research.

## 1. Introduction

Providing a standardized, efficient, and cost-effective model for building food systems and applying it to the classification framework of emerging dietary patterns is a highly challenging and important task. Food classification systems, whether nutrient- or processing-based, such as the NOVA system, may not fully capture the complexity of modern diets, including fortified and processed plant-based foods [1]. The emergence of new dietary patterns has prompted substantial investment in reconstructing food classification systems. For instance, at least seven processing-based frameworks have been developed to support epidemiological and experimental research [2,3,4]. These developments have also motivated researchers to explore ways of adapting and applying existing systems to enhance efficiency, ensure data consistency, and reduce labor and cost burdens.

The standardization, harmonization, and FAIRization of food data, aimed at organizing and applying such data across diverse domains, has emerged as a new frontier, particularly in linking food, environmental, nutritional, and health-related information through integrated modeling approaches [5]. To address this need, the European Food Safety Authority (EFSA) developed FoodEx2, a system integrating food classification and dietary data from national consumption databases across EU countries. By using standardized food descriptors, FoodEx2 reduces labor and time for cross-country data harmonization [6,7,8]. It consists of eight hierarchical modules—MTX, Reporting, Zoonoses, Feed, Exposure, VetDrugRes, Botanicals, and FeedAddExpo—offering a comprehensive framework for managing food-related data across regulatory and research domains.

In Taiwan, a harmonized, food description-incorporated, food classification system (HFDFC) has been widely applied as a standardized and scalable framework in dietary research [9]. Nevertheless, the HFDFC system remains limited in its capacity to address emerging dietary patterns, such as the plant-based diet (PBD), which pose growing challenges to contemporary dietary research. To date, no food classification framework has incorporated food descriptors specifically designed to support the identification and analysis of plant-based diets.

The plant-based diet (PBD) is an emerging dietary pattern recognized as important for promoting human health and protecting the environment through decreased carbon emissions [10,11,12]. The PBD focuses on foods such as vegetables, fruits, whole grains, legumes, and nuts and leverages bioactive compounds abundant in plants, such as catechins, anthocyanins, polyphenols, and phytosterols, to effectively prevent a range of chronic non-communicable diseases, including type 2 diabetes, hypertension, dyslipidemia, atherosclerosis, and cancer. In 2016, the plant-based dietary index (PDI) was proposed to assess the types of plant-based foods consumed and the amount of animal-based foods included in the PBD [13]. Based on consumer dietary intake data, dietary patterns, and dietary status are assessed using the overall PDI (O-PDI), healthy PDI (H-PDI), and less healthy PDI (Lh-PDI). The plant-based diet index (PDI) concept extends beyond the evaluation of plant-based foods alone, encompassing animal-based foods as well as unhealthy dietary components [14,15]. These indices have been widely applied in nutrition and medical research [16]. When analyzing diet and nutritional status, the nutrient-rich food (NRF) index is a key indicator used by consumers to distinguish between nutrient-dense and minimally processed foods. Its effectiveness in promoting healthy diets has been widely validated [17]. For example, nutrients associated with chronic disease risks, such as sodium, sugar, saturated fat, and trans fats, as well as beneficial nutrients such as protein and fiber, can serve as key points for analysis, deepening the understanding of the relationship between disease and dietary intake [17,18,19]. Therefore, this study aimed to develop a plant-based diet food classification (PBDFC) system based on the HFDFC framework. After applying the PBDFC to Taiwan’s nutrition survey data, the study combined the plant-based diet index (PDI) and the Nutrient-Rich Food (NRF) index to conduct a descriptive analysis of the distribution of health-related variables within the population.

## 2. Materials and Methods

### 2.1. HFDFC System

The HFDFC system comprises a classification system, a description system, and a coding system and incorporates secondary data obtained from the Nutrition and Health Survey conducted in Taiwan. It reflects the dietary intake of the Taiwanese population and includes not only 17 level-one food groups—(1) wholegrain and mixed grains; (2) dry beans and nuts; (3) fats and oils; (4) poultry and poultry products; (5) livestock and livestock products; (6) fish and seafood; (7) eggs; (8) dairy; (9) fruits; (10) vegetables; (11) sugar and confections; (12) drinks; (13) wine; (14) seasonings; (15) composite food, soups, and other categories; (16) infant foods; and (17) health foods—but also 67 level-two sub-groups, 199 level-three sub-groups, and 131 level-four sub-groups.

The description system provides comprehensive information on various facets of food, such as intake, food sources, processed products, cooking methods, manufacturers (brands), food additives, specialty foods, and main ingredients. Each facet comprises several specific descriptions. For example, the facet “processed products” includes descriptions such as canned products, pickled products, smoked products, fermented products, and other processed food products. The coding system serves to integrate the classification and description systems, linking food categories with their corresponding descriptive attributes [9,20].

The HFDFC system enhances research applicability through two key features as follows: (1) food facets can be combined with food classification to build thematic databases, such as intake databases or nutrient databases, and (2) food descriptions can be converted into food classifications and vice versa. This bidirectional conversion is particularly useful in the analysis of composite foods. By referencing recipe databases, the facet “main ingredients” can be used to describe the individual ingredients used within a composite dish [20]. For example, in “beef noodles”, both beef and noodles are included under the facet of main ingredients while also being listed as items in the food classification system.

### 2.2. Research Framework

**Figure 1:**
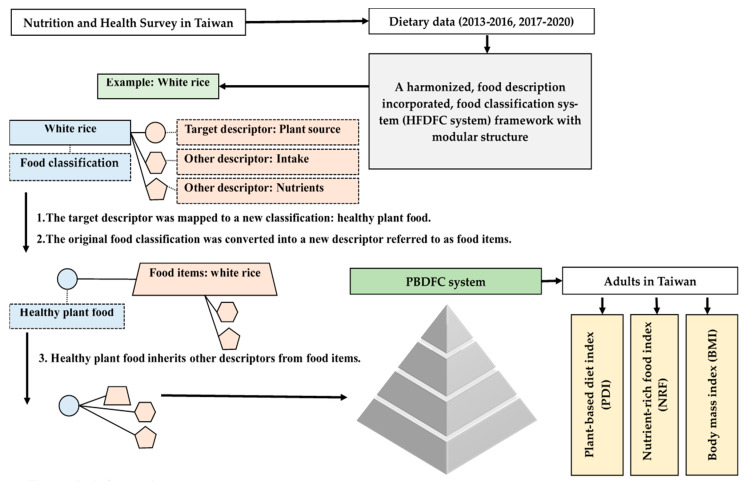
Study framework.

Figure 1 illustrates this study’s framework, detailing the transformation of the Harmonized, Food Description Incorporated, Food Classification system (HFDFC) into the Plant-Based Diet Food Classification system (PBDFC). Dietary data from the 2013–2016 and 2017–2020 Nutrition and Health Survey in Taiwan, covering participants aged 20–70 years, were incorporated into the HFDFC’s four-level structure. This system categorizes detailed food items, such as white rice, and accompanies each with facets like intake, nutrients, and crucial food sources.

The “food sources” facet specifically includes plant, animal, and other origins. The PBDFC system was then developed through a two-step transformation based on literature-derived PBD definitions. First, food source descriptions were mapped to new food groups, such as healthy plant foods, animal foods, and less healthy foods. Second, the original detailed food classifications were converted into individual food item descriptors (e.g., white rice). Crucially, the newly defined healthy plant food classification inherits other relevant descriptors (e.g., intake, nutrients) directly from these original food items. This ensures the transformed PBDFC system retains comprehensive dietary information for subsequent analysis. This reclassification created new food categories, while specific items like white rice maintained their identity. The resulting PBDFC system enabled the evaluation of plant-based diet index (PDI), nutrient-rich food index (NRF), and body mass index (BMI) in Taiwanese adults.

### 2.3. Plant-Based Dietary Index

Based on international studies referenced in the development of the PBDFC and PDI in this study, foods were categorized into three groups: healthy plant foods, less healthy plant foods, and animal foods. The purpose of the PDI is to assess the extent to which plant-based foods are emphasized within an individual’s overall diet. Therefore, all three categories are included in the index to reflect the balance between plant- and animal-based food intake. Animal foods are incorporated as negative scoring components, which allows the PDI to capture variations in adherence to plant-based dietary patterns [13,21,22]. First, the intake amounts for each food category were summed for each participant and then divided into quartiles (Q) according to the distribution across all samples. Second, the O-PDI, H-PDI, and Lh-PDI scores were calculated [13,23]. In scoring the O-PDI, both healthy plant foods and less healthy foods were assigned positive scores, with participants in Q1 receiving 1 point and those in Q5 receiving 5 points. In contrast, animal foods were reverse scored, with Q1 receiving 5 points and Q5 receiving 1 point. In scoring the H-PDI, healthy plant foods were positively scored, whereas less healthy foods and animal foods were reverse scored. In scoring the Lh-PDI, less healthy foods were positively scored, whereas healthy plant foods and animal foods were reverse scored. Finally, the total O-PDI, H-PDI, and Lh-PDI scores were obtained by summing the scores for individual foods. The total score ranged from 14 to 70, with higher scores indicating a greater adherence to the dietary pattern defined by each index. The O-PDI, H-PDI, and Lh-PDI were categorized into tertiles, with T1, T2, and T3 representing the lowest, middle, and highest tertiles, respectively.

### 2.4. Nutrition-Rich Food Index

The NRF index can be used to identify foods with high nutritional value and limit nutrients that are detrimental to health. A commonly used model, such as the NRF9.3 model, evaluates dietary quality based on a framework that includes nine nutrients to encourage (ENs) and three nutrients to limit (LNs), providing a comprehensive assessment of overall nutritional quality [24].

We calculated the NRF index for foods using the Formulas (1) and (2) shown below, where n/m represents the number of ENs or LNs, and NRF_nm__Ec refers to the NRF value per 100 calories. An NRF value > 0 indicates a positive nutritional benefit. The calculation is based on the recommended dietary allowance (RDA) for each nutrient, as well as the maximum recommended value (MRV) for saturated fat and sodium [25]. EI represents energy intake.

NRF_nm_ = ∑_1-n_ (EN/RDA) − ∑_1-m_ (LN/MRV) (1)

NRF_nm__Ec = ((NRFnm × 100/Intake)/EI) × 100 (2)

### 2.5. Statistical Analysis

Statistical analyses were performed using SAS version 9.4 (SAS Institute Inc., Cary, NC, USA) to evaluate the nutritional intake status of Taiwanese adults aged 20–70 years, based on secondary data from a survey. The Mann–Whitney U test was used for inferential statistical analysis.

## 3. Results

### 3.1. Food Classification Framework and Scoring Criteria for PDI and NRF132

As shown in Table 1, Among the 17 level-one food groups originally defined in the HFDFC system, 14 groups relevant to this study were selected and reclassified based on food source descriptions, and transformed into the PBDFC food classification. These were further categorized into three major food types: healthy plant foods, less healthy foods, and animal-based foods. Specifically, healthy plant foods include whole grains and mixed grains, dry beans and nuts, fruits, vegetables, and plant fats and oils; less healthy foods include sugar and confections, drinks, and refined grains; and animal foods include poultry and poultry products, livestock and livestock products, fish and seafood, eggs, dairy, and animal fats and oils. In the O-PDI, H-PDI, and Lh-PDI, each food group is assigned a positive (+) or negative (−) score based on its health effect. Nrf_Ec132 serves as an indicator of the overall nutritional intake quality and includes 13 ENs (fiber, protein, calcium, iron, potassium, magnesium, zinc, and vitamins A, C, E, B_1_, B_2_, and B_12_) and two LNs (saturated fat and sodium).

### 3.2. Distribution of Plant-Based Diet Indices, Nutritional Quality, and BMI Across Age Groups

Table 2 shows that during both 2013–2016 and 2017–2020, individuals aged 46–70 years had significantly higher O-PDI and H-PDI scores and lower Lh-PDI scores (*p* < 0.0001), indicating higher intake of healthy plant foods, than other age groups. Body mass index (BMI) was also significantly higher in the 46–70 age group than in other age groups, with values of 24.8 ± 2.75 kg/m^2^ during 2013–2016 and 24.98 ± 3.29 kg/m^2^ during 2017–2020 (both *p* < 0.0001). Regarding Nrf_Ec132, individuals aged 46–70 years had significantly higher values than those aged 20–45 years in both periods, with median values of 24.39 vs. 20.78 during 2013–2016 and 24.63 vs. 18.62 during 2017–2020 (*p* < 0.0001), indicating better overall dietary nutrient density. Overall, with increasing age, the participants exhibited a healthier plant-based dietary pattern and higher nutritional quality. Despite the upward trend in BMI, a generally better dietary structure was observed.

Table 3 shows the relationships between the T1 and T3 of the O-PDI, H-PDI, and Lh-PDI with BMI and Nrf_Ec132. During both the 2013–2016 and 2017–2020 periods, for the O-PDI and H-PDI, Nrf_Ec132 was significantly higher in T3 than in T1 across all age groups (*p* < 0.0001). For the Lh-PDI, Nrf_Ec132 was significantly lower in T3 than in T1 (*p* < 0.0001).

For the O-PDI, during the 2013–2016 period, Nrf_Ec132 was significantly higher in T3 than in T1 in the 20–45 age group, with a value of 22.22 (*p* = 0.0301), and BMI was significantly lower in T3 than in T1 in the 46–70 age group, with a median value of 24.55 kg/m^2^ (*p* = 0.0010). Regarding the H-PDI, BMI was significantly lower in T3 than in T1 in both the 20–45 and 46–70 age groups, with median values of 24.64 (*p* = 0.0021) and 24.82 (*p* = 0.0147), respectively. For the Lh-PDI, during the 2017–2020 period, BMI was significantly lower in T3 than in T1 in the 20–45 age group, with a median value of 22.39 kg/m^2^ (*p* = 0.0002); however, in the 46–70 age group, BMI was slightly higher in T3 than in T1, with a median value of 25.11 kg/m^2^ (*p* = 0.0403).

### 3.3. Changes in Food Intake Composition Across Plant-Based Diet Index Categories

**Figure 2:**
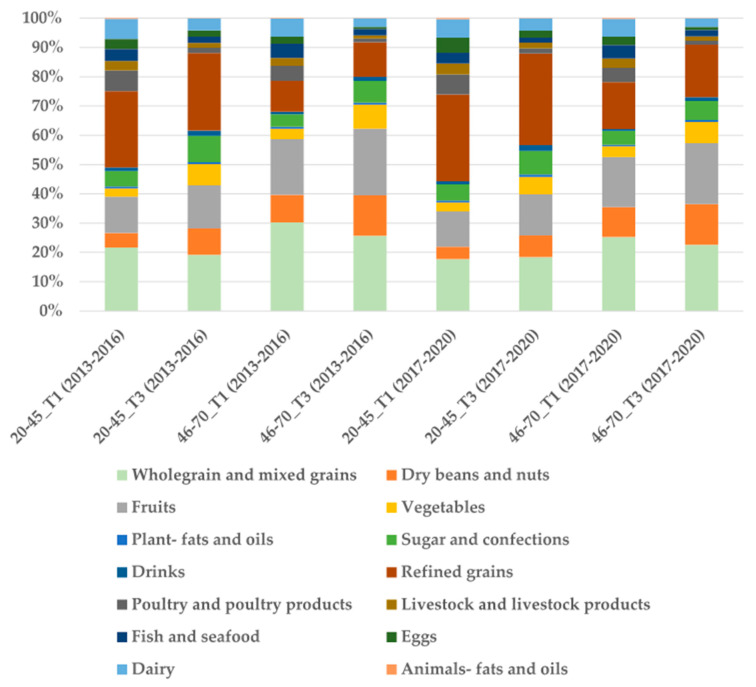
Nutrient intakes and food group proportional intakes of participants in O-PDI.

**Figure 3:**
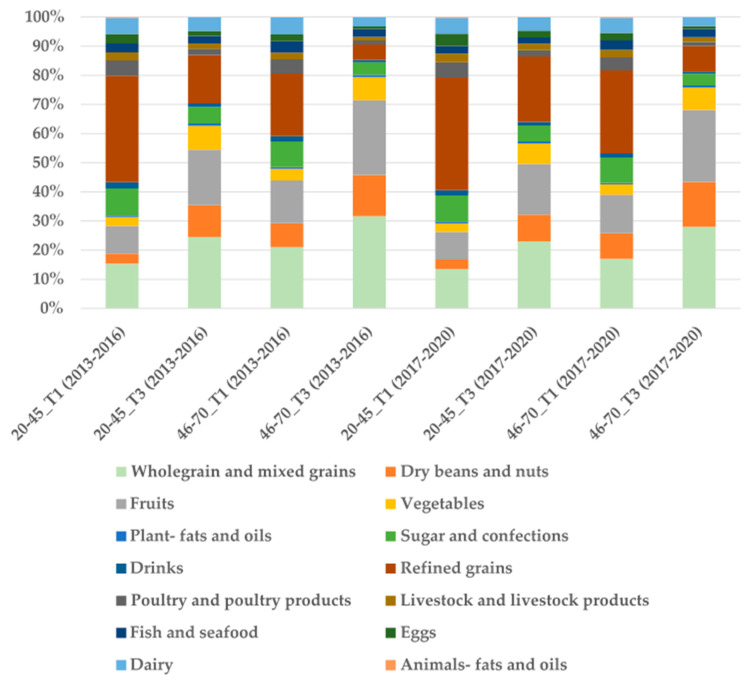
Nutrient intakes and food group proportional intakes of participants in H-PDI.

**Figure 4:**
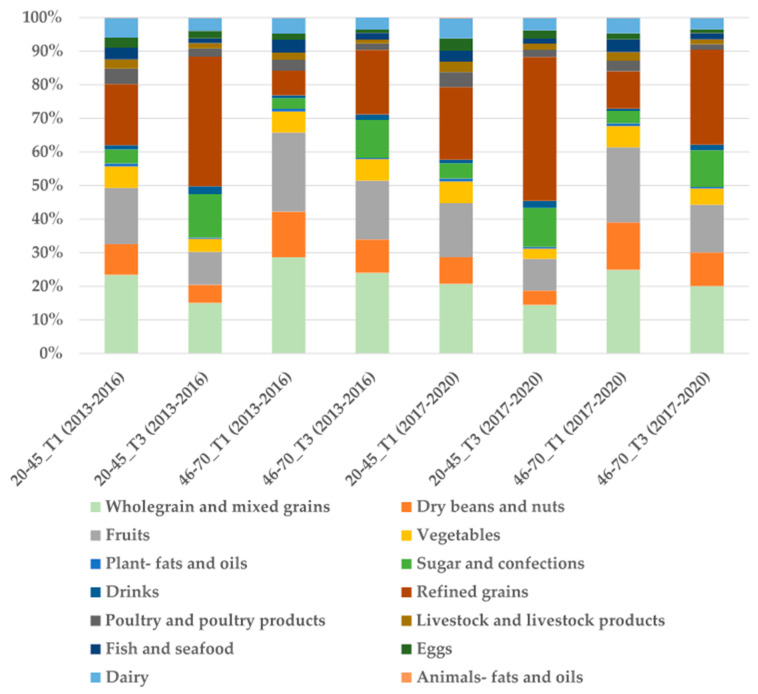
Nutrient intakes and food group proportional intakes of participants in Lh-PDI.

Figure 2, Figure 3 and Figure 4 show the percentage changes in food intake across different food categories in the O-PDI, H-PDI, and Lh-PDI. In the O-PDI (Figure 2), the intake proportion of healthy plant foods was significantly higher in T3 than in T1, being highest in the 46–70 age group during 2013–2016 (71.14%) and lowest in the 20–45 age group during 2017–2020 (37.67%) in T1. The intake proportion of less healthy foods was highest in the 20–45 age group in T3 during 2017–2020 (41.41%, with refined grains accounting for 31.35%) and lowest in the 46–70 age group in T1 during 2013–2016 (15.66%, with refined grains accounting for 10.53%). The intake proportion of animal foods was highest in the 20–45 age group in T1 during 2017–2020 (26.06%) and lowest in the 46–70 age group in T3 during the same period (9.04%). The differences across groups were statistically significant (*p* < 0.001).

Figure 3 shows that in the H-PDI group, the intake proportion of healthy plant foods was significantly higher in T3 than in T1 (with the highest at 80.15% and the lowest at 29.80%). The intake proportion of less healthy foods was lower in T3 than T1 (with the lowest at 10.48%, of which refined grains accounted for 5.30%, and the highest at 49.48%, of which refined grains accounted for 38.69%). Regarding animal foods, the lowest intake was observed in T3 in the 46–70 age group during 2013–2016 (9.37%), whereas the highest intake was in T1 in the 20–45 age group during 2017–2020 (20.71%). All differences were statistically significant (*p* < 0.01).

Figure 4 shows that in the Lh-PDI group, the intake proportion of healthy plant foods was lowest in T3 in the 20–45 age group during 2017–2020 (31.72%) and highest in T1 in the 46–70 age group during 2013–2016 (72.86%). The intake proportion of less healthy foods was highest in T3 in the 20–45 age group (56.58%, with refined grains accounting for 42.85%) and lowest in T1 in the 46–70 age group (11.30%, with refined grains accounting for 7.27%). The intake proportion of animal foods was highest in T1 in the 20–45 age group during 2017–2020 (20.66%) and lowest in T3 in the 46–70 age group (9.53%). The differences between T1 and T3 were statistically significant (*p* < 0.01).

## 4. Discussion

Our findings indicate that with increasing age, the participants’ O-PDI and H-PDI increased significantly, whereas their Lh-PDI decreased significantly, reflecting a shift toward a healthier overall dietary pattern, with a tendency to consume more healthy plant foods and reduce the intake of less healthy foods. This trend is also evident in the Nrf_Ec132, with the 46–70 age group significantly outscoring the 20–45 age group. On the other hand, investigations have found dietary patterns rich in healthy plant foods are significantly associated with slower aging processes [26] and suggests a positive relationship between the PBD and cognitive function in older adults [22]. Overall, O-PDI and H-PDI scores are negatively associated with mortality risk, whereas Lh-PDI scores are positively associated with mortality risk [27]. Furthermore, research indicates that maintaining a healthy PBD may help reduce mortality risk among socioeconomically disadvantaged groups, with the protective benefits being particularly pronounced in those with the lowest socioeconomic status [27].

This study further compares the NRF index and BMI across different PDI categories. In both the O-PDI and H-PDI groups, Nrf_Ec132 was significantly higher in T3 than in T1, indicating a positive association between overall and health-oriented PBDs and dietary nutrient density. Notably, in the H-PDI group, BMI was significantly lower in T3 than in T1 in the 46–70 age group across both time periods, supporting a potential beneficial relationship between healthy PBDs and body weight control. A similar decreasing trend in BMI was also observed across age groups in the O-PDI group during the 2013–2016 period. However, it is worth noting that BMI was significantly higher in this age group than in others, and similar findings have been reported in other studies [28]. Conversely, in the Lh-PDI group, the median Nrf_Ec132 values were significantly lower in T3 than in T1 across all age groups and time periods, reflecting a notable decrease in overall nutrient density with increasing intake of less healthy foods. Furthermore, in the 20–45 age group, BMI was significantly lower in T3 than in T1; however, in the 46–70 age group, BMI was significantly higher in T3 than in T1. A possible explanation for these divergent findings is a potential interaction between the Lh-PDI and BMI across different age groups, warranting further investigation into the underlying mechanisms.

In both the O-PDI and H-PDI groups, the intake proportion of healthy plant foods was significantly higher in T3 than in T1, with whole grains and mixed grains, fruits, legumes, and vegetables being the primary contributors. This dietary pattern aligns with recommended nutritional guidelines. In terms of the nutritional adequacy of PBD, the literature highlights key nutrients of concern, including vitamin B_12_, iron, zinc, calcium, vitamin D, and omega-3 fatty acids [29]. Furthermore, in addition to total intake, incorporating quality-based indices offers a clearer picture of the health benefits of plant-based diets. Unrefined grains, legumes, fruits, and vegetables are key to chronic disease prevention. Emphasizing quality helps guide healthier food substitutions and improves overall diet [16].

Conversely, the intake proportions of less healthy based foods (such as refined grains and sugar-sweetened beverages) and animal foods were significantly lower in T3 than in T1. In contrast, in the Lh-PDI group, T3 showed the highest intake proportion of less healthy foods, with refined grains accounting for up to 42.85%, whereas the intake proportions of healthy plant foods and animal foods were markedly lower. The Lh-PDI, characterized by a high consumption of refined grains, sweets, desserts, and sugar-sweetened beverages, is positively associated with weight gain [30] and BMI [31].

To the best of our knowledge, this is the first study to establish a PBDFC system adapted from the food source descriptors in the HFDFC system. In contrast, FoodEx2 derives new classifications from the FoodEx2 Matrix (MTX), which organizes food items based on their names and coding structures tailored to specific regulatory or research objectives [32]. Building on this contrast, we aim to propose an alternative model for constructing food classification systems and highlight the flexibility of the HFDFC framework in supporting emerging dietary patterns through the derivation of the PBDFC. Moreover, the food source descriptors already established in the HFDFC, such as those identifying processed products [9], can be further applied to reflect classification systems like NOVA. This allows for the potential development of an HFDFC-NOVA system with four categories as follows: unprocessed/minimally processed foods, culinary ingredients, processed foods, and ultra-processed foods in future.

This study has some limitations. First, we did not adjust for potential confounding factors such as socioeconomic status, education, physical activity, and supplement use, which may have affected dietary quality and BMI. Second, although the PBDFC system provides a structured way to classify plant-based diets, its fixed criteria for “healthy” and “less healthy” foods may not fully reflect the variety of modern plant-based products. Lastly, while PDI and NRF132 are common tools for assessing diet quality, PDI may not clearly separate ultra-processed plant-based foods, and NRF132 does not consider harmful compounds such as acrylamide, polycyclic aromatic hydrocarbons, or artificial additives.

## 5. Conclusions

This study demonstrated the feasibility of adapting the HFDFC system for standardizing dietary data and classifying emerging dietary patterns. By integrating PDI, BMI, and Nrf_Ec132, the analysis revealed age-related differences in dietary quality and health indicators. While the findings support the utility of this approach for assessing plant-based diets, the lack of control for socioeconomic and lifestyle factors may have influenced the results. This study may serve as a reference for future public health and nutrition research.

## Figures and Tables

**Table 1:** Food classification and scoring criteria used for PBDFC, PDI, and NRF132.

HFDFC Classification	PBDFC Classification	OPDI	HPDI	LhPDI	RDA of NRF132			Note
					Nutrients	Male	Female	
Wholegrain and mixed grains	Healthy plant foods	+	+	−	Fiber	24 g	20 g	Encourage nutrients
Dry beans and nuts		+	+	−	Protein	70 g	60 g	
Fruits		+	+	−	Vitiman A	600 µg	500 µg	
Vegetables		+	+	−	Vitiman C	100 mg	100 mg	
Plant-fats and oils		+	+	−	Vitiman E	12 mg	12 mg	
Sugar and confections	Less healthy foods	+	−	+	Ca	1000 mg	1000 mg	
Drinks		+	−	+	Iron	15 mg	10 mg	
Refined grains;		+	−	+	Potassium	2800 mg	2500 mg	
Poultry and poultry products	Animal foods	−	−	−	Magnesium	360 mg	310 mg	
Livestock and livestock products		−	−	−	Zinc	15 mg	12 mg	
Fish and seafood		−	−	−	B_1_	1.2 mg	0.9 mg	
Eggs		−	−	−	B_2_	1.3 mg	1.0 mg	
Dairy		−	−	−	B_12_	2.4 µg	2.4 µg	
Animals-fats and oils		−	−	−	Saturated fat	2300 mg	1800 mg	Limit nutrients
					Na	2300 mg	2300 mg	

**Table 2:** Characteristics of the study population by survey period and age group.

Survey Period	Age Groups	*N*	OPDI	HPDI	LhPDI	BMI	Nrf_Ec132
			Mean (SD)				Median
2013–2016	20–45	1865	43.48 (5.84)	42.5 (6.33)	45.07 (7.49)	22.06 (3.85)	20.78
	46–70	2577	45.1 (5.61)	47.92 (6.37)	43.49 (6.91)	24.8 (2.75)	24.39
2017–2020	20–45	1897	42.77 (5.64)	41.51 (6.11)	45.18 (7.01)	22.91 (4.58)	18.62
	46–70	3107	44.83 (5.74)	46.85 (6.39)	43.11 (6.74)	24.98 (3.29)	24.63

Note: Between-group differences by age (20–45 vs. 46–70 years) within each survey period were all statistically significant (*p* < 0.0001; *T* test or Mann–Whitney U test).

**Table 3:** BMI and Nrf_Ec132 in PDI groups.

		2013–2016			2017–2020		
Age: 20–45	Variables	T1 (*n* = 582)	T3 (*n* = 911)	*p* Value	T1 (*n* = 555)	T3 (*n* = 853)	*p* Value
O-PDI	BMI	21.79	22.22	0.0301 *	22.76	23.10	0.1827
	Nrf_Ec132	17.35	24.61	<0.0001 ^+^	13.59	22.44	<0.0001 ^+^
H-PDI	BMI	22.21	21.89	0.1273	23.17	22.77	0.1188
	Nrf_Ec132	18.16	22.96	0.0036 ^+^	13.98	21.77	<0.0001 ^+^
Lh-PDI	BMI	22.22	21.95	0.1887	23.31	22.39	0.0002 *
	Nrf_Ec132	25.17	15.03	<0.0001 ^+^	24.88	12.31	<0.0001 ^+^
**Age: 46–70**	**Variables**	**T1 (*n* = 870)**	**T3 (*n* = 1052)**	***p* value**	**T1 (*n* = 894)**	**T3 (*n* = 1393)**	***p* value**
O-PDI	BMI	24.96	24.55	0.0010 *	25.07	24.87	0.1771
	Nrf_Ec132	20.55	26.71	<0.0001 ^+^	18.65	29.51	<0.0001 ^+^
H-PDI	BMI	25.06	24.64	0.0021 *	25.18	24.82	0.0147 *
	Nrf_Ec132	22.02	26.16	0.0558 ^+^	21.21	26.98	0.0002 ^+^
Lh-PDI	BMI	24.91	24.68	0.0787	24.82	25.11	0.0403 *
	Nrf_Ec132	29.06	17.02	<0.0001 ^+^	30.09	17.63	<0.0001 ^+^

Note: * *T* Test; ^+^ Mann–Whitney U test.

## Data Availability

The original contributions presented in this study are included in the article. Further inquiries can be directed to the corresponding author.
